# Dopamine-transporter levels drive striatal responses to apomorphine in Parkinson's disease

**DOI:** 10.1002/brb3.115

**Published:** 2013-03-22

**Authors:** Luca Passamonti, Maria Salsone, Nicola Toschi, Antonio Cerasa, Marco Giannelli, Carmelina Chiriaco, Giuseppe Lucio Cascini, Francesco Fera, Aldo Quattrone

**Affiliations:** 1 Istituto di Scienze Neurologiche (ISN), Consiglio Nazionale delle Ricerche88100, Catanzaro, Italia; 2 Dipartimento di Scienze Mediche e Chirurgiche, Università degli Studi “Magna Graecia”88100, Catanzaro, Italia; 3 Dipartimento di Biopatologia e Diagnostica per Immagini, Università degli Studi di Roma “Tor Vergata”00133, Roma, Italia; 4 Unità operativa di Fisica Medica, Azienda Ospedaliero-Universitaria Pisana56126, Pisa, Italia; 5 Dipartimento di Medicina Sperimentale e Clinica, Università degli Studi “Magna Graecia”88100, Catanzaro, Italia

**Keywords:** Cognition, DAT, dopamine-agonist, fMRI, Parkinson's disease, working memory

## Abstract

Dopaminergic therapy in Parkinson's disease (PD) can improve some cognitive functions while worsening others. These opposite effects might reflect different levels of residual dopamine in distinct parts of the striatum, although the underlying mechanisms remain poorly understood. We used functional magnetic resonance imaging (fMRI) to address how apomorphine, a potent dopamine agonist, influences brain activity associated with working memory in PD patients with variable levels of nigrostriatal degeneration, as assessed via dopamine-transporter (DAT) scan. Twelve PD patients underwent two fMRI sessions (Off-, On-apomorphine) and one DAT-scan session. Twelve sex-, age-, and education-matched healthy controls underwent one fMRI session. The core fMRI analyses explored: (1) the main effect of group; (2) the main effect of treatment; and (3) linear and nonlinear interactions between treatment and DAT levels. Relative to controls, PD-Off patients showed greater activations within posterior attentional regions (e.g., precuneus). PD-On versus PD-Off patients displayed reduced left superior frontal gyrus activation and enhanced striatal activation during working-memory task. The relation between DAT levels and striatal responses to apomorphine followed an inverted-U-shaped model (i.e., the apomorphine effect on striatal activity in PD patients with intermediate DAT levels was opposite to that observed in PD patients with higher and lower DAT levels). Previous research in PD demonstrated that the nigrostriatal degeneration (tracked via DAT scan) is associated with inverted-U-shaped rearrangements of postsynaptic D2-receptors sensitivity. Hence, it can be hypothesized that individual differences in DAT levels drove striatal responses to apomorphine via D2-receptor-mediated mechanisms.

## Introduction

The key role of dopaminergic drugs in modulating cognitive functions of patients with Parkinson's disease (PD) has been consistently demonstrated (Brooks 2006; Cools 2006; Kehagia et al. 2010). In particular, dopamine replacement therapy (i.e., l-Dopa) has been shown to enhance working memory, a cognitive process depending on prefrontal cortex (PFC) and striatal circuits (Lewis et al. 2005; Cools 2006). However, dopaminergic drugs may also have detrimental effects on different neuropsychological functions such as reversal learning (Swainson et al. 2000). Recently, increased susceptibility to distraction has also been demonstrated in PD patients treated with l-Dopa and/or dopamine agonists, a group of drugs that directly stimulate dopaminergic receptors (Cools et al. 2010).

In order to reconcile these contradictory findings, it has been proposed that the effects of dopaminergic drugs depend on task demands as well as the residual level of endogenous dopamine in nigrostriatal and ventral tegmental area (VTA) PFC circuits (“dopamine overdose hypothesis”) (Cools 2006; Kehagia et al. 2010; de la Fuente-Fernandez 2012). Previous evidence also showed that the relation between dopaminergic neurotransmission and PFC function follows an inverted-U-shaped model (Williams and Goldman-Rakic 1995; Arnsten and Goldman-Rakic 1998). In other words, a drug that restores the function of a dopamine-depleted circuit might simultaneously overdose, and thus impair the function of a different network with a less severe dopaminergic deficit (Cools 2006; Kehagia et al. 2010).

Nonetheless, the complex relation between dopaminergic drugs and cognition in PD is far from being fully characterized and several questions remain open. For example, it is still unclear how dopamine agonists impact on cognition in PD and whether the brain responses associated with specific neuropsychological functions might be influenced by individual levels of nigrostriatal degeneration.

This study investigated the effects of apomorphine, a potent and fast-acting dopamine agonist, on neural activity during working memory in PD patients with variable levels of nigrostriatal degeneration, as assessed by dopamine-transporter (DAT) imaging. Previous research in animal models of PD combined radio-isotopic and functional magnetic resonance imaging (fMRI) to investigate the relations between dopaminergic damage (tracked via DAT scan), D2 receptor sensitivity (measured with raclopride, a dopamine agonist), and blood oxygenation level–dependant (BOLD) response after infusion of apomorphine (Nguyen et al. 2000; Zhang et al. 2000, 2001, 2006). These studies demonstrated that dopaminergic damage (i.e., reduced DAT levels) was associated with enhanced striatal BOLD response to apomorphine (Nguyen et al. 2000; Zhang et al. 2000, 2001, 2006) and increased D2 receptor sensitivity (i.e., enhanced raclopride binding; Nguyen et al. 2000).

Apomorphine is a nonselective dopamine agonist, although it exhibits a very high affinity for the D2 receptor family, and particularly the D4 receptor (Millan et al. 2002). At low doses (i.e., ∼0.004 mg/kg), apomorphine reduces the dopamine release within the striatum via the inhibitory D2 receptors located presynaptically (i.e., on the nigrostriatal terminals) (Montoya et al. 2008; Schellekens et al. 2010). In contrast, at the higher (10-fold) doses used for PD treatment (i.e., ∼0.04 mg/kg), apomorphine mainly stimulates the postsynaptic D2 receptors expressed by striatal neurons (Bowron 2004; LeWitt 2004).

However, the way apomorphine influences cognition in PD remains unclear, and previous studies have produced mixed results. In particular, two initial experiments in PD patients with intermediate disease stages showed that apomorphine lengthens reaction times during working memory and increases the latency of event-related potentials during an odd-ball task (Ruzicka et al. 1994; Costa et al. 2003). In contrast, two later studies in patients with more advanced forms of PD reported no effect of apomorphine infusion on a series of neuropsychological tests (Alegret et al. 2004; De Gaspari et al. 2006).

Our hypothesis was that the action of apomorphine on striatal responses associated with working memory depends on the level of nigrostriatal degeneration, which we quantified via DAT imaging. Given the inverted-U-shaped relation between dopaminergic neurotransmission and PFC function, we also hypothesized that brain responses to apomorphine followed a nonlinear model (Williams and Goldman-Rakic 1995; Arnsten and Goldman-Rakic 1998). In particular, apomorphine was expected to overdose VTA–PFC circuits in patients with less severe dopaminergic deficit, as recently proposed by a staging model of cognitive deficits in PD (de la Fuente-Fernandez 2012).

## Participants and Methods

### Participants

Sixteen PD patients and 13 sex-, education-, and age-matched healthy controls (HCs; no neuropsychiatric diseases and normal structural MRI of the brain) gave their written informed consent to participate in this study, approved by the Ethics Board of the University “Magna Graecia” of Catanzaro. A junior (M. S.) and a senior (A. Q.) neurologist, both blind to other results, made the diagnosis of PD in accordance to the United Kingdom Parkinson's Disease Society Brain Bank criteria (Hughes et al. 1992). Age of onset, disease duration, and severity of symptoms, as assessed by the Unified Parkinson's Disease Rating Scale (UPDRS) and Hoehn–Yahr stage, were recorded. Additional inclusion criteria for PD patients were (1) no dementia according to the *DSM-IV* (American Psychiatric Association 1994), (2) no use of psychoactive drugs (e.g., benzodiazepines, antidepressants) during 1 month preceding the experiment, (3) no use of caffeine within 24 h before the experiment, (4) no history of smoking, (5) no major depression according to the *DSM-IV* criteria (American Psychiatric Association 1994), (6) right handedness, as assessed by the Edinburgh Handedness Inventory (Oldfield 1971), and (7) normal structural MRI of the brain.

In all participants, a trained neuropsychologist (C. C.) collected the following neuropsychological measures: (1) executive control (Frontal Assessment Battery, Modified Card Sorting Test) (Nelson 1976; Iavarone et al. 2004), (2) short- and long-term verbal memory (Rey Auditory Verbal Learning Test; Rey 1958), (3) attention and working memory (Digit Span Forward and Backward; Wechsler 1981), (4) verbal fluency and language comprehension (Controlled Oral Word Association Test, Token Test; De Renzi and Vignolo 1962; Benton et al. 1994), and (5) visual–spatial skills (Judgment of Line Orientation; Benton et al. 1978). Although none of the participants met the criteria for major depressive episodes and other psychiatric disorders, we further investigated the presence of depressive and anxiety symptoms using the Beck Depression Inventory and the Hamilton Rating Scale Anxiety, respectively (Hamilton 1959; Beck and Steer 1987). In PD patients, these cognitive functions were only evaluated Off-therapy given the short half-life of apomorphine (see next section). Overall, the neuropsychological session lasted approximately 1 h.

Demographic and neuropsychological data were analyzed using independent two-sample *t*-tests within SPSS (Statistical Package for Social Sciences, http://www-01.ibm.com/software/analytics/spss/).

### Apomorphine test in PD patients

To prevent vomiting and/or nausea, patients were administered domperidone (60 mg/daily) for 48 h before the apomorphine test (Bowron 2004). Fourteen hours prior to scanning, all medications except domperidone were withdrawn. PD patients attended two distinct sessions (i.e., Off- and On-apomorphine). The apomorphine test was executed either at the first or second fMRI session in a counterbalanced order across patients (sessions were separated by at least 1 day). This protocol ensured a complete drug clearance in case apomorphine was administered at the first session (apomorphine half-life is ∼45 min; Bowron 2004; LeWitt 2004).

Apomorphine was subcutaneously injected 10 min before scanning at the dose of 0.04 mg/kg (mean dose ∼3 mg). This schedule and dosage allow us to study apomorphine effects on cognitive functions approximately at drug peak dose and under a strong postsynaptic pharmacological action (Bowron 2004; LeWitt 2004). Motor symptoms were assessed via UPDRS before and after apomorphine injection. To account for nonspecific drug effects on arousal, patients were also asked to report their arousal level via a specific questionnaire (Mackay et al. 1978) before and after apomorphine injection.

### fMRI task

Participants executed a modified version of a verbal working-memory paradigm that has been previously validated in PD patients and that evokes robust activations within the PFC and striatum (Lewis et al. 2003, 2004).

There were three types of trials: (1) high-load working memory: subjects were instructed to remember a string of six uppercase letters presented for 2 sec and followed by a 3-sec delay of blank screen. Next, a lowercase probe letter was displayed for 2 sec and subjects were asked, within this time-window, to press a button when the probe matched any of the letters previously displayed in the string. Alternatively, no response was required. A 1-sec delay of blank screen concluded the trial that lasted 8 sec in total; (2) medium-load working memory: trials were identical to previous ones except for a string that contained three letters intermixed to three abstract symbols (#). The position of letters and symbols within the string was counterbalanced across trials; (3) low-load working memory: as before, but the string contained one letter and five symbols.

Four trials of each type were grouped in a block lasting 32 sec. The task included 18 blocks (six high-, six medium-, and six low-load working-memory blocks) alternated, in a pseudorandom order, to six fixation blocks (12 sec each) during which subjects passively viewed a cross at the center of the screen (total task duration: 10 min, 48 sec). To familiarize with the task design, participants practiced a short version of the paradigm that contained a different set of stimuli from that used during the fMRI session.

Stimuli were projected onto a back projection screen throughout a LCD video projector while reaction times (RT) and responses at each trial were recorded via an MRI compatible fiber optic button box response controlled by LabVIEW (National Instruments, Austin, TX, http://www.ni.com/labview/i/).

RT and response accuracy were entered in separate analyses of variance (ANOVA) models assessing: (1) the main effect of task; (2) the main effect of group (PD-Off, controls); (3) the group by task interaction (PD-Off, Controls × high-, medium-, and low-load working memory); (4) the main effect of treatment (PD-Off, PD-On); (5) the treatment by task interaction (PD-Off, PD-On × high-, medium-, and low-load working memory); and (6) linear and quadratic interactions between treatment and DAT levels in PD patients (PD-Off, PD-On × DAT levels).

### MRI acquisition

MRI scanning was performed on a 3.0 Tesla Unit with an 8-channels head coil (Discovery MR-750, General Electric, Milwaukee, WI). Head movements during scanning were minimized using comfortable foam pads around participants' head. A T1-weighted structural scan was obtained (368 sagittal slices, 1-mm thickness each; repetition time 9.2 msec; echo time 3.7 msec; voxel size 1 × 1 × 1 mm) to allow the cortical and subcortical segmentation procedures that were necessary for the quantitative analysis of DAT level (see Cortical and subcortical segmentation and Quantitative DAT imaging of the striatum sections). fMRI data were acquired with echo planar images (EPI) sensitive to the BOLD contrast (35 axial slices, 3-mm thickness each; repetition time 2000 msec; echo time 25 msec; voxel size 3 × 3 × 3 mm).

### DAT acquisition

PD patients underwent the 123-iodine-fluoropropyl-single-photon emission computed tomography scan (123-I-FP-SPECT) on a separate day within 2 weeks before or after the fMRI sessions. Of note, PD patients were Off-therapy during the DAT acquisition (i.e., 12 h prior to DAT scanning, all medications for PD were withdrawn).

Patients received perclorate (1000 mg) 30 min before scanning to block the thyroid uptake of free radioactive iodine. Brain imaging was performed 3 h after the administration of 200 MBq of 123-I-FP (GE-Amersham, Eindhoven, the Netherlands) using a dual-headed gamma camera (Infinia Hawkeye, General Electric, Milwaukee, WI) equipped with low-energy, high-resolution collimators. Scans were acquired with a photopeak window centered on 159 keV ± 10% with a 128 × 128 image matrix (zoom factor: 1.5, 40 sec per view and 2 × 64 views). The slice thickness was 2.95 mm. Patients were carefully positioned in the gamma camera, with their meato-orbital axis in a transverse plane to reduce reorientation during reconstruction, in a special head holder that allowed a minimal rotation distance. Images were reconstructed using a Butterworth filter (cutoff 0.5 and order 6). Chang's correction method's was used to compensate for attenuation using a coefficient, μ, of 0.11 cm^−1^.

### Cortical and subcortical segmentation

This procedure was performed to allow a quantitative analysis of the DAT levels in the caudate and putamen of PD patients as described in Quantitative DAT imaging of the striatum section. Cortical and subcortical reconstruction and volumetric segmentation of each patient's structural T1-weighted structural scan were performed with the Freesurfer image analysis suite (http://surfer.nmr.mgh.harvard.edu/) (Fischl 2012). Briefly, this includes removal of nonbrain tissue using a hybrid watershed/surface deformation procedure, automated Talairach transformation, segmentation of the subcortical white-matter and deep gray-matter volumetric structures, intensity normalization, tessellation of the gray-matter–white-matter boundary, automated topology correction, and surface deformation following intensity gradients to optimally place the gray/white and gray/cerebrospinal fluid borders at the location where the greatest shift in intensity defines the transition to the other tissue class.

A patient-specific region of interest (ROI) located in the occipital cortex (ROI_occ_) was successively created by merging the ROIs obtained from the cortical segmentation stream delineating the lateral occipital, lingual, cuneus, and pericalcarine regions. ROIs delineating the putamen and caudate nuclei were obtained from the subcortical segmentation stream. All ROIs were defined in each patient's native T1 space and were used in the calculation of region- and patient-specific, background-subtracted DAT uptake ratio (see Quantitative DAT imaging of the striatum section). We employed an accurate segmentation procedure which accounts for interpatient differences both in cortical and in subcortical anatomy in order to increase accuracy as compared with procedures which use standard striatal atlases as well as patient-specific segmentation procedures which involve applying relatively simple affine transformations from standard to patient space when delineating the occipital cortex.

### Quantitative DAT imaging of the striatum

For every patient, the DAT image containing the raw DAT-binding potential signal (BP_raw_) was registered to his or her T1 image using the command-line tool FLIRT (http://www.fmrib.ox.ac.uk/fsl/; Jenkinson et al. 2002), thereby transforming each subject's DAT image into his or her native T1 space. We used a normalized mutual information-driven, 12-degree of freedom affine transformation followed by sinc interpolation in order to compensate for inhomogeneous distortions artifacts in T1 and SPECT images. All further analyses were performed in this space, thereby avoiding the combination of multiple smoothing and/or resampling steps which would have resulted by first transforming the images into a standard atlas space. A reference raw DAT-BP_ref_ value originating from unbound or nonspecifically bound 123-I-FP was calculated by overlaying each subjects' ROI_occ_ onto his or her registered DAT image and averaging within this region (the occipital cortex is assumed to be devoid of DATs) (Scherfler and Nocker 2009). This value was assumed to represent the nonspecific radioligand-binding compartment (Cline et al. 1992; Laruelle et al. 1994; Helmich et al. 2011). The signal related through specific DAT binding was then estimated following the ROI-based approach described by Scherfler and Nocker (2009) and as recommended by the European Nuclear Medicine Association (https://www.eanm.org). Specifically, the background-subtracted striatal uptake ratio (DAT-BP_ND_ index, which can be assumed to relate directly to the binding potential at equilibrium between a compartment with specific binding and a compartment representing nonspecifically bound or nondisplaceable and free activity) was calculated in each voxel as DAT-BP_ND_ index = (BP_raw_ − BP_ref_)/BP_ref_. The resulting images were averaged within each patient's putamen and caudate nucleus by overlaying the corresponding ROIs, hence obtaining a normalized measure of specific DAT binding in these regions.

Next, ANOVA models were run within SPSS to assess (1) significant differences in the DAT-BP_ND_ values between the caudate and putamen, bilaterally (i.e., main effect of the striatal region); (2) significant differences in the DAT-BP_ND_ values between the left and right hemisphere (i.e., main effect of the hemisphere); and (3) any region by hemisphere interaction (caudate, putamen DAT-BP_ND_ values × left, side hemisphere).

Finally, individual DAT-BP_ND_ values in the left and right striatum were tested for possible correlations with PD duration and, importantly, used as separate regressors for fMRI analyses (see fMRI analyses section).

### fMRI analyses

fMRI data were analyzed using SPM8 (http://www.fil.ion.ucl.ac.uk/spm/). Images were initially realigned to the first scan through rigid body transformations to correct for head movements, next normalized to the standard template in the Montreal Neurological Institute (MNI) space using linear and nonlinear transformations, and finally smoothed with a Gaussian kernel of full width at half maximum of 8 mm.

A random effect model was implemented using a two-stage process (first- and second-level) allowing inferences about the general population from which participants were drawn. For each subject, we used a generalized linear model (GLM) to evaluate effects of task parameters on BOLD activations (Friston et al. 1994). The model included four experimental factors (high-, medium-, low-load working-memory trials, and fixation baseline) and six realignment parameters which were included as covariates of no interest in order to account for residual motion-related variance. Low-frequency signal drift was removed using a high-pass filter (cutoff = 128 sec) and an autoregressive modeling (AR [1]) of temporal autocorrelations was applied. At the first level, subject-specific contrast images were generated for each working-memory load condition versus baseline. Each working-memory load versus baseline contrast was then entered into second-level GLM ANOVAs to obtain SPM-*F* maps that investigated: (1) the main effect of task; (2) the main effect of group (PD-Off, Controls); (3) the group by task interaction (PD-Off, Controls × high-, medium-, and low-load working memory); (4) the main effect of treatment (PD-Off, PD-On); and (5) the treatment by task interaction (PD-Off, PD-On × high-, medium-, and low-load working memory). Furthermore, to account for possible effects of behavioral variability on brain activations, analyses (2), (3), (4), and (5) were repeated including RT and accuracy as variables of no interest.

Of note, we also tested for linear and quadratic interactive effects between medication and DAT-BP_ND_ values on striatal BOLD responses in the PD group. The SPM model included two separate regressors for each patient: (1) the DAT-BP_ND_ values (testing for linear effects); (2) the square of these values (testing for quadratic functions). This way, it is possible to investigate linear fits (excluding quadratic ones) and vice versa (i.e., testing for quadratic effects factoring out linear ones). This method has been used before in fMRI studies exploring linear and nonlinear relations between drug effects on clinical variables in PD patients (Rowe et al. 2008). The same method was also used to study linear and quadratic effects of disease duration.

Analyses exploring activations within the whole brain were thresholded at *P* < 0.05, family-wise error (FWE), whole-brain correction. In addition, given our strong a priori hypotheses on specific PFC and striatal regions, we employed a ROI approach. ROIs included the anterior cingulate cortex (ACC); the superior, middle, and inferior frontal gyrus (SFG, MFG, IFG); the caudate; and putamen and were created using the “aal.02” atlas (http://marsbar.sourceforge.net/) (Tzourio-Mazoyer et al. 2002). The statistical threshold for ROI analyses was set at *P* < 0.05, FWE, small volume correction (svc) (Worsley et al. 1996; Friston 1997). Because 12 ROIs (six on the left, six on the right) with different size were defined, we treated them as separate hypotheses and further adjusted the significance for multiple comparison testing using Dunn–Sidak correction (Howell et al. 2007).

Finally, for explorative purposes only, we also report brain regions not predicted a priori but that met a threshold of *P* < 0.001, uncorrected, >10 contiguous voxels.

## Results

### Participants

Three PD patients and one HC subject displayed head movements >3 mm during at least one fMRI session; hence, their data were not included in the final analysis. An additional PD patient was excluded because she presented high scores on the Beck Depression Inventory and Hamilton Rating Scale for Anxiety (23 and 17, respectively). As a result, data from 12 PD patients and 12 HCs were available for the final analysis.

Table 1 summarizes the main demographic, clinical, and neuropsychological measures of PD patients and HC included in the analyses. The majority of patients (9/12) were taking l-Dopa and presented a mild form of PD (Hoehn–Yahr = 1.8 ± 0.4). As expected in PD, seven patients showed prevalent motor symptoms at right limbs while the remaining five patients were most affected at left limbs.

**Table 1:** Demographic, clinical, and neuropsychological characteristics of Parkinson's disease (PD) patients and healthy controls

		PD patients (*n* = 12)		Healthy controls (*n* = 12)		
				
Demographic and clinical data	Measure	Mean	SD	Mean	SD	*P* value^1^
Demographic data	Sex	Three females, nine males		Five females, seven males		
	Age (years)	59.6	6.8	60.2	7.3	0.84
	Education (years)	10.8	4.0	9.5	4.4	0.45
Clinical data	Age of onset (years)	56.0	6.9	–	–	–
	Disease duration (months)	36.7	27.7	–	–	–
	Hoehn–Yahr stage	1.8	0.4	–	–	–
	UPDRS-ME Off score	21.5	8.4	–	–	–
	UPDRS-ME On score	12.3	4.2	–	–	–
	l-Dopa daily equivalent dose (mg)^2^	454.7	316.1	–	–	–
	Motor response to apomorphine (%)^3^	16.7	9.4	–	–	–
Cognitive function examined	Neuropsychological test used	PD patients (*n* = 12) 4		Healthy controls (*n* = 12)		
Executive control	Frontal assessment battery	15.4	1.8	15.2	1.8	0.81
	MCST correct answers	5.1	1.3	5.5	1.1	0.42
	MCST perseverative errors	1.2	1.3	1.0	1.9	0.84
Short-term verbal memory	RAVLT (immediate recall)	36.6	9.4	41.2	6.8	0.18
Long-term verbal memory	RAVLT (delayed recall)	7.2	2.7	8.3	2.2	0.31
Attention and working memory	Digit span forward	5.9	0.8	5.9	0.8	0.86
	Digit span backward	3.7	0.7	3.7	0.8	1.00
Verbal fluency	Controlled oral word association test	28.2	7.9	25.5	8.4	0.43
Language comprehension	Token test	31.4	1.4	32.2	1.3	0.14
Visual–spatial skills	Judgment of lines orientation	23.0	5.4	23.0	5.2	0.97
Anxiety	Hamilton Anxiety Rating Scale	9.6	4.9	8.6	6.1	0.66
Depression	Beck Depression Inventory	11.4	7.0	8.9	7.2	0.40

SD, standard deviation; UPDRS-ME, Unified Parkinson's Disease Rating Scale Motor Examination; MCST, Modified Card Sorting Test; RAVLT, Rey Auditory Verbal Learning Test.

1 *P* values obtained from two-sample independent *t*-tests.

2 l-Dopa daily equivalent dose was calculated following the protocol described in Tomlinson et al., Mov. Disord. 2010, Table 2.

3 The percentage (%) motor response to apomorphine was calculated as follows: (UPDRS-ME Off [i.e., after 12 h of withdrawn from any dopaminergic drug] − UPDRS-ME On [i.e., ∼20 min after apomorphine subcutaneous injection])/UPDRS-ME Off × 100.

4 Neuropsychological tests in PD patients were administered Off-treatment.

Of note, PD patients showed comparable scores to HCs on a series of tests evaluating cognitive and emotional measures such as executive control, short- and long-term verbal memory, attention and working memory, verbal fluency, language comprehension, visual–spatial skills, anxiety, and depression levels (Table 1).

Finally, the level of arousal in PD patients did not change after apomorphine injection (Off: mean = 8.4± 4.1; On: mean = 8.1 ± 3.7).

### fMRI behavioral performances

As expected, there was an highly significant main effect of working-memory load for both RT and accuracy (RT: *F*_df(10)_ = 45.97, *P* < 0.0001; accuracy: *F*_df(10)_ = 28.4, *P* < 0.0001) that depended on lengthened RT and reduced accuracy for progressively higher working-memory loads.

For both RT and accuracy, we found no main effect of group (PD-Off, HCs) (RT: *F*_df(11)_ = 0.4, *P* = 0.5; accuracy: *F*_df(11)_ = 1.3, *P* = 0.3, respectively) nor a group by task interaction (PD-Off, HCs × high-, medium-, and low-load working memory) (RT: *F*_df(10)_ = 1.3, *P* = 0.3; accuracy: *F*_df(10)_ = 2.2, *P* = 0.2).

No main effect of treatment (PD-Off, PD-On) was found for RT (*F*_df(11)_ = 1.2, *P* = 0.28) while a trend was detected for accuracy (*F*_df(11)_ = 3.6, *P* = 0.08). Although nonsignificant, this latter result was due to reduced accuracy levels in PD patients under apomorphine, relative to Off-medication, during all working-memory loads.

Furthermore, there was no treatment by task interaction (PD-Off, PD-On × high-, medium-, and low-working-memory load) for both RT and accuracy (RT: *F*_df(10)_ = 0.3, *P* = 0.72; accuracy: *F*_df(10)_ = 0.7, *P* = 0.49).

Finally, no linear or quadratic effects were found in the interaction between accuracy, treatment, and DAT-BP_ND_ values in PD patients (*F*'s < 2.2; *P*'s > 0.1).

### DAT-imaging results

When analyzing whether DAT-BP_ND_ values differed between striatal regions (i.e., caudate, putamen) and between the left and right hemisphere, we found a strongly significant main effect of the striatal area (*F* = 16.9; *P* < 0.003) that depended on lower DAT-BP_ND_ values in the putamen relative to those in the caudate (i.e., left caudate = 1.36 ± 0.47, right caudate = 1.45 ± 0.62, left putamen = 1.12 ± 0.43, right putamen = 1.26 ± 0.59).

In contrast, there was no main effect of the hemisphere (i.e., left, right) (*F* = 1.1; *P* = 0.30), nor any region by hemisphere interaction (i.e., caudate, putamen DAT-BP_ND_ values × left, right hemisphere) (*F* = 0.5; *P* = 0.47).

**Figure 1:**
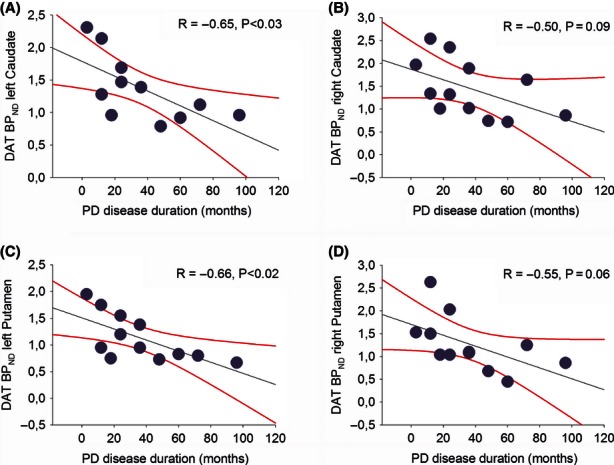
Correlation analyses between the average dopamine-transporter (DAT)-binding values for the bilateral striatum (caudate and putamen), and the duration (in months) of Parkinson's disease (PD). Please note that due to the extremely close DAT values in the left caudate (1.386934 and 1.392076, respectively) and right putamen (1.084890 and 1.101013, respectively) of two PD patients with 36 months of disease duration, two data points appear indistinguishable in Figure 1A and D.

Of note, PD duration was negatively correlated with DAT-BP_ND_ values in the left striatum (left caudate: *R* = −0.65, *P* < 0.03; left putamen: *R* = −0.66, *P* < 0.02) (i.e., patients with longer disease duration displayed significantly lower left striatal DAT-BP_ND_ values) while a borderline effect was found in the right striatum (right caudate: *R* = −0.50, *P* = 0.09; right putamen: *R* = −0.55, *P* = 0.06) (Fig. 1).

Overall, these DAT-imaging results revealed the typical pattern of dopaminergic degeneration in PD (i.e., greater dopaminergic loss in putamen compared to the caudate) and confirmed previous findings showing that disease duration correlates with the level of dopamine loss in the striatum (Antonini et al. 1995). No main effect of the hemisphere, or a region by hemisphere interaction, was found but this obviously depended on the presence of a similar number of PD patients with a prevalently left-sided (*n* = 5) and right-sided (*n* = 7) disease.

### fMRI results

**Figure 2:**
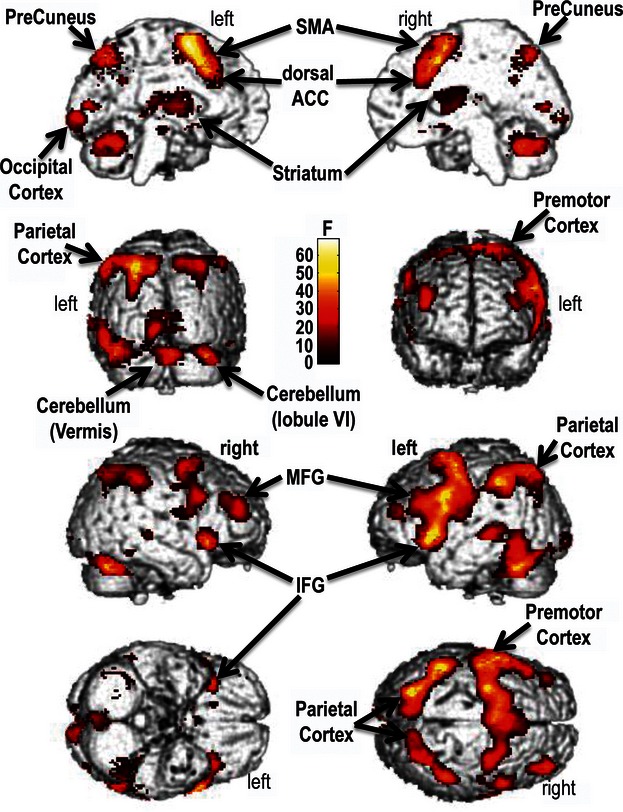
Main effect of task. The brain regions shown are those where the BOLD activity progressively increases as a function of higher working-memory loads. The color bar represents *F* statistics. Maps are thresholded at *P* < 0.05, family-wise error (FWE), whole-brain correction. SMA, supplementary motor area; MFG, middle frontal gyrus; IFG, inferior frontal gyrus; ACC, anterior cingulate cortex; BOLD, blood oxygenated level dependant.

The ANOVA investigating the main effect of task revealed several regions within and outside ROIs that showed progressively increased activations as a function of the working-memory load (*F*'s_df(66)_ > 15, *P*'s < 0.05, FWE, whole-brain correction) (Fig. 2).

The ANOVA exploring the main effect of group (PD-Off, HCs) showed greater activations in the left middle occipital cortex (left: *x*, −16; *y*, −100; *z*, 0; *F* = 18.23, *P* < 0.001) and right cuneus (*x*, 22; *y*, −90; *z*, 28; *F* = 13.72, *P* < 0.001, uncorrected) in PD patients versus controls. A significant group by task interaction was also detected in the right precuneus (*x*, 22; *y*, −82; *z*, 34; *F* = 14.63, *P* < 0.001, uncorrected) and left thalamus (*x*, −14; *y*, −28; *z*, 14; *F* = 9.05, *P* < 0.001, uncorrected). These latter findings were driven by increased BOLD response in PD-Off patients versus HCs only during high-load working-memory trials.

**Figure 3:**
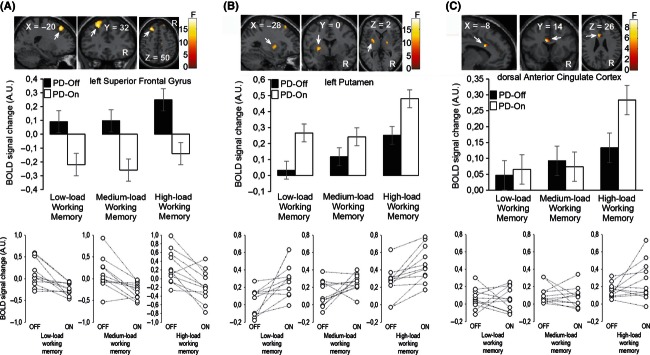
(A) Main effect of treatment. The left superior frontal gyrus displayed reduced response in Parkinson's disease (PD) patients under apomorphine (PD-On) compared with patients without medication (PD-Off) during all working-memory loads. (B) Main effect of treatment. The left putamen showed enhanced activations in PD-On compared with PD-Off during all working-memory loads. (C) Treatment × Task interaction. The dorsal anterior cingulate cortex displayed increased response in PD-On compared with PD-Off only during high-load working-memory trials. The color bars represent *F* statistics. Coordinates (*x*, *y*, and *z*) are in the Montreal Neurological Institute (MNI) space. Middle and bottom panels show data plots from the brain region displayed immediately above (mean BOLD signal change ±SE, and individual data plots, respectively). BOLD, blood oxygenated level dependent; A.U., arbitrary unit; R, right hemisphere. Only for display purposes, maps are thresholded at *P* < 0.005, uncorrected, but results are significant at *P* < 0.05, family-wise error (FWE), small volume correction (svc).

A significant treatment effect (PD-Off, PD-On) was found in the left superior frontal gyrus (SFG) and left putamen (*P*'s < 0.05, FWE, svc) (Fig. 3A and B). Specifically, PD-On versus PD-Off patients showed reduced left SFG activity and increased response in the putamen. Furthermore, a treatment by task interaction revealed that PD-On versus PD-Off patients displayed greater dorsal ACC (dACC) activity only during high-load working-memory trials (Fig. 3C) (*P* < 0.05, FWE, svc). Finally, very similar results were obtained when the analyses were repeated including both RT and accuracy as variables of no interest (*F*'s_df(66)_ > 8, *P*'s < 0.05, FWE, svc).

**Figure 4:**
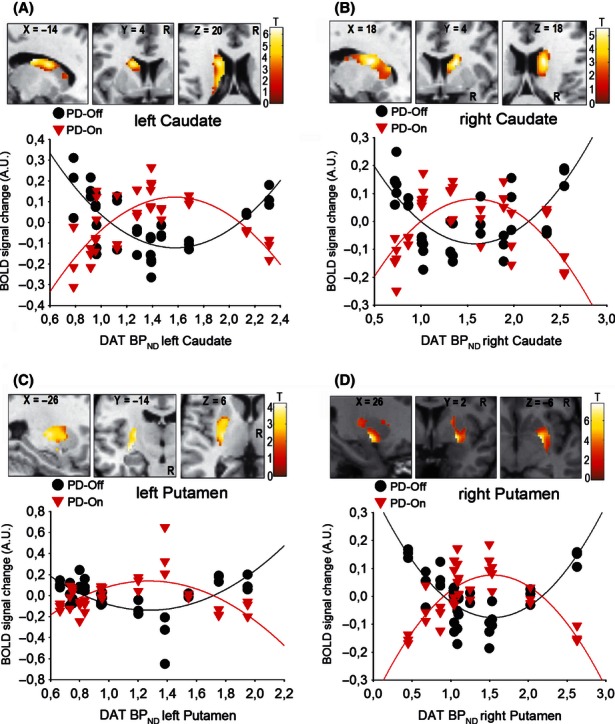
(A–D) Nonlinear interactions between treatment (Off-, On-apomorphine), striatal response during low-, medium-, high-load working memory, and dopamine transporter (DAT)-BP_ND_ values in patients with Parkinson's disease (PD). In PD-Off, the relation between the striatal BOLD activity and DAT-BP_ND_ values follows a U-shaped model; in contrast, the same relation follows an inverted*-*U shape model in PD-On. The color bars represent *T* statistics. For display purposes, maps are thresholded at *P* < 0.005, uncorrected, but results are significant at *P* < 0.05, family-wise error (FWE), small volume correction (svc). Coordinates (*x*, *y*, and *z*) are in the Montreal Neurological Institute (MNI) space. BOLD, blood oxygenated level dependent; A.U., arbitrary unit; R, right hemisphere.

When testing for linear and nonlinear interactions between treatment (PD-Off, PD-On) and DAT-BP_ND_ values in PD patients, we found a significant quadratic (but not linear) effect in the bilateral striatum (*P*'s < 0.05, FWE, svc). In particular, the orientation of a U-shaped relation between the striatal response and DAT-BP_ND_ values under Off-treatment was reversed by apomorphine (i.e., it became inverted-U) (Fig. 4A–D). Similar findings were obtained for extra-striatal PFC ROIs (*P*'s < 0.05, FWE, svc; Fig. S1). Essentially, the effect of apomorphine on striatal and PFC activity in PD patients with intermediate DAT-BP_ND_ values was opposite to the effect observed in patients with higher and lower DAT-BP_ND_ values. Finally, no statistically significant linear or quadratic effects were found for disease duration in all ROIs at *P* < 0.05, FWE, svc.

## Discussion

We used multimodal neuroimaging to study how individual differences in nigrostriatal degeneration, as quantified by DAT scan, influenced BOLD responses to apomorphine, a potent and fast-acting dopamine agonist.

We found that DAT-BP_ND_ levels guided the striatal and PFC responses to apomorphine in PD patients during all working-memory loads. In particular, the apomorphine effect in PD patients with intermediate dopaminergic depletion was opposite to that found in patients with higher and lower dopaminergic depletion (i.e., patients with longer and shorter disease duration, respectively).

Consistent with some previous data, apomorphine tended to impair behavioral performance during working memory in all PD patients, regardless of the residual dopamine level (Ruzicka et al. 1994; Costa et al. 2003). However, only a trend effect of treatment was found for accuracy (*P* = 0.08), and this may depend on our smaller sample size (*n* = 12) compared with those commonly used to assess the behavioral effects of dopaminergic drugs (e.g., *n* ∼20) (Costa et al. 2009). Further studies with larger sample sizes are necessary to identify the precise amount of apomorphine stimulation that leads to cognitive dysfunctions. We also acknowledge that additional research is necessary to investigate how apomorphine influences cognition in PD patients with greater disease severity and longer disease duration that those reported here.

Nonetheless, it is important to point out that this study was designed to explore how apomorphine influenced working memory in PD at a neural rather than behavioral level. To this end, fMRI is a sensitive tool which can reveal subtle effects of drugs on brain responses, even before the occurrence of noticeable behavioral findings. In fact, apomorphine modulated neural responses independently from its behavioral effects, and this was demonstrated by the stability of the results when fMRI analyses assessing the main effect of treatment were repeated including RT and accuracy as variables of no interest. Overall, our data extend the knowledge about the neural mechanisms of apomorphine in PD by showing that this potent dopamine agonist increased striatal response and reduced SFG activation during working memory.

The enhanced striatal response to apomorphine might depend on the super-sensitivity of postsynaptic D2 receptors. There is evidence, in animal models of PD, that lesioning dopaminergic neurons causes reduced DAT-BP_ND_ values, increased D2 receptor binding, and increased BOLD response to apomorphine in the striatum (Nguyen et al. 2000). Comparative research has also suggested that this enhanced striatal BOLD response to apomorphine may indirectly reflect the state of postsynaptic D2 receptors (i.e., sensitivity and/or number) (Zhang et al. 2000, 2006). Although the sensitivity and/or number of D2 receptors were not measured in this study, we speculate that the progressive nigrostriatal degeneration in PD induced a D2 receptor super-sensitivity state which, in turn, guided the abnormal striatal responses to apomorphine during all working-memory loads. However, it remains to be explained why we found an inverted-U-shaped relation between DAT-BP_ND_ values and the brain responses to apomorphine. We hypothesize at least two, not mutually exclusive, explanations for this finding.

First, there is clear in vitro evidence that the number of striatal D2 receptors follows an inverted-U curve after lesioning dopaminergic neurons (i.e., the number of receptors continue to rise until the ∼100th day after the dopaminergic damage; next, it gradually reverts to normal levels, which are reached after ∼500 days in total) (Todd et al. 1996).

Second, an inverted-U-shaped relation between D2 receptors number and/or sensitivity and disease progression has been also observed in vivo, in PD patients at different stages (Antonini et al. 1994, 1995; Ichise et al. 1999). In particular, patients with initial or advanced PD display normal D2 receptor number and/or sensitivity, while patients with intermediate disease progression show increased D2 receptors number and/or sensitivity (Antonini et al. 1994, 1995; Ichise et al. 1999). Other studies have also demonstrated that D2 receptors in PD return to normal levels after treatment and remain stable over the course of the disease (Guttman and Seeman 1985; Guttman et al. 1986). This variability may depend on differential levels of exposure to chronic dopaminergic therapy that might normalize the receptor number and/or sensitivity (i.e., patients with advanced PD would have been more exposed to chronic dopaminergic therapy when compared with patients with intermediate PD stages) (Alexander et al. 1993). However, it is also possible that molecular mechanisms independent from drug therapy intervene to reduce the D2 receptor number and/or sensitivity over time. There is indeed evidence that the number and/or sensitivity of D2 receptors decreases in Parkinsonian monkeys with chronic nigrostriatal lesion even if they did not receive dopaminergic therapy (Decamp et al. 1999). Nonetheless, differences in treatment duration in our PD patients may have played a role in determining the sensitivity of D2 receptors and thus the heterogeneity of their brain responses to apomorphine.

It is also noteworthy that apomorphine decreased activation of the SFG, a specific PFC region linked to stimulus manipulation during working memory (du Boisgueheneuc et al. 2006). SFG is linked to basal-ganglia circuits involved in filtering irrelevant information during working memory (Moustafa et al. 2008; Baier et al. 2010); hence, apomorphine might indirectly alter the SFG function via dopaminergic receptors in the striatum. Our finding that DAT striatal levels modulated BOLD responses to apomorphine in SFG during all working-memory loads support this hypothesis. Alternatively, apomorphine might influence dopaminergic receptors on cortical neurons within the SFG itself. This possibility is supported by previous research in behaving monkeys showing that excessive levels of D1 receptor stimulation reduce delay-related firing of PFC neurons and erode the tuning of their responses during working memory (Vijayraghavan et al. 2007). In line with a recent staging model of executive dysfunctions and mental fatigue in PD (de la Fuente-Fernandez 2012), it is also possible that apomorphine stimulation “overdosed” the direct VTA-PFC dopaminergic pathways via D4 receptors, a D2 receptor family expressed in the neocortex and implicated in the pathophysiology of a range of neuropsychiatric disorders (Oak et al. 2000; Wang et al. 2002).

Two other PFC areas (i.e., the inferior frontal gyrus, IFG, and the dACC) showed a significant modulation by the striatal DAT levels and apomorphine therapy. The IFG has been consistently associated with response inhibition, a key neuropsychological function during working-memory tasks that require response inhibition (Aron and Poldrack 2005, 2006; Aron 2011). In contrast, the dACC has been linked to error and conflict monitoring, two other fundamental processes to execute a wide range of cognitive paradigms (van Veen and Carter 2002; Hester et al. 2004; Nee et al. 2011). It is also important to note that apomorphine effects related to DAT levels were consistently observed for all working-memory loads (i.e., high-, medium-, and low-working-memory loads). Overall, this suggests a broad influence of apomorphine on PFC physiology that is independent from cognitive demands.

Finally, two additional points should be discussed. First, we did not assess the brain effects of apomorphine in our HCs because of the emetic of the high doses of drug employed in PD patients (Bowron 2004; LeWitt 2004). Furthermore, even if we had used apomorphine at low doses in HCs, the interpretation of the group by treatment interaction would have been limited by the fact that apomorphine at those doses activates presynaptic rather than postsynaptic D2 receptors (the latter is the case at the high doses employed in PD). Nonetheless, we enrolled HCs to examine the main effect of group (PD-Off, HCs). This analysis showed that PD patients Off-medication, relative to controls, displayed greater activations in the cuneus, precuneus, and thalamus, a group of regions previously implicated in attentional and working-memory processes (LaBar et al. 1999). BOLD hyperactivations associated with normal behavioral performances have been previously described in PD and other neurological patients and may represent functional compensations and brain plasticity effects (Passamonti et al. 2009, 2011; Hughes et al. 2010). Alternatively, they may result from reduced “focusing” within working memory and attentional circuits (Mattay et al. 2002; Helmich et al. 2009).

Second, it could be argued that our results were partially driven by apomorphine effects on other neurochemical systems involved in arousal (i.e., apomorphine also modulates noradrenergic receptors, although to a lesser extent than dopaminergic ones) (LeWitt 2004). Although this possibility cannot be completely ruled out, our patients did not report significant changes in the arousal state after apomorphine injection. Furthermore, a medication by task interaction was found in the dACC, demonstrating that at least part of the neural effects induced by apomorphine were specific for high-load working-memory trials.

In conclusion, our research offers strong support in demonstrating that the combination of fMRI and quantitative DAT imaging predicted individual differences in brain responses to a dopaminergic challenge. In the future, additional studies with larger sample sizes may open new possibilities for developing multiparametric brain markers (e.g., diffusional kurtosis imaging [Giannelli et al. 2012]) that can be used to personalize pharmacological therapies according to the specific needs of PD patients.

## Supporting Information

**Figure S1.** Nonlinear interactions between treatment (Off-, On-apomorphine), prefrontal cortex (PFC) response during low-, medium-, high-load working memory, and dopamine transporter (DAT)-BPND values in patients with Parkinson's disease (PD). In PD-Off, the relation between the PFC BOLD activity and DAT-BPND values follows a U-shaped model; in contrast, the same relation follows an inverted-U shape model in PD-On. The color bars represent T statistics. For display purposes, maps are thresholded at *P* < 0.005, uncorrected, but results are significant at *P* < 0.05, familywise error (FWE), small volume correction (svc). Coordinates (*x*, *y*, and *z*) are in the Montreal Neurological Institute (MNI) space. BOLD, blood oxygenated level dependent.
