# Supplementary figures and images for: Dopamine-transporter levels drive striatal responses to apomorphine in Parkinson's disease
Source: Brain Behav. 2013 Mar 22;3(3):249–62. doi: 10.1002/brb3.115 (PMC3683285; doi:10.1002/brb3.115)

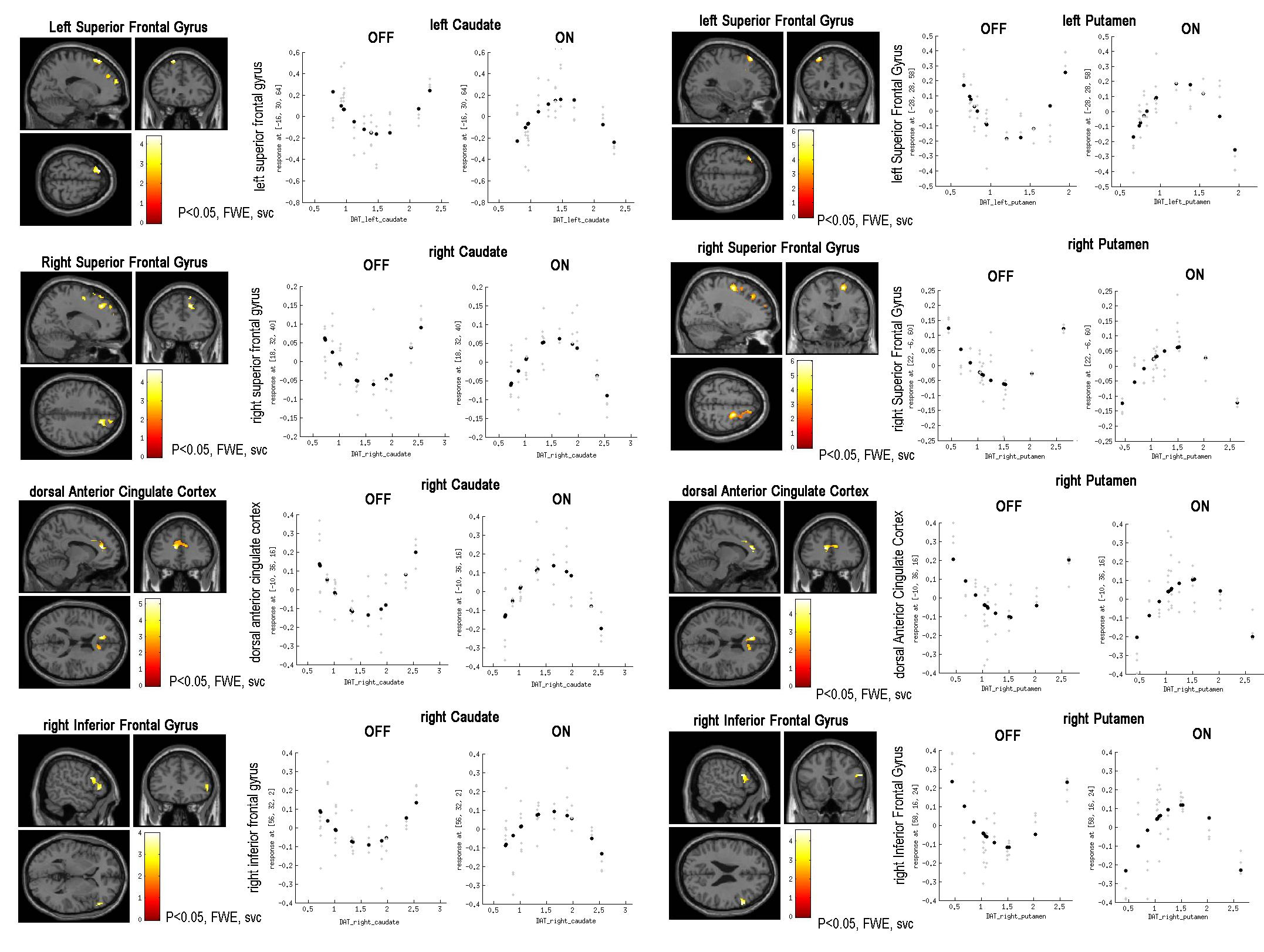

Supplement: Supplementary file 1 [file brb30003-0249-SD1.tif]
